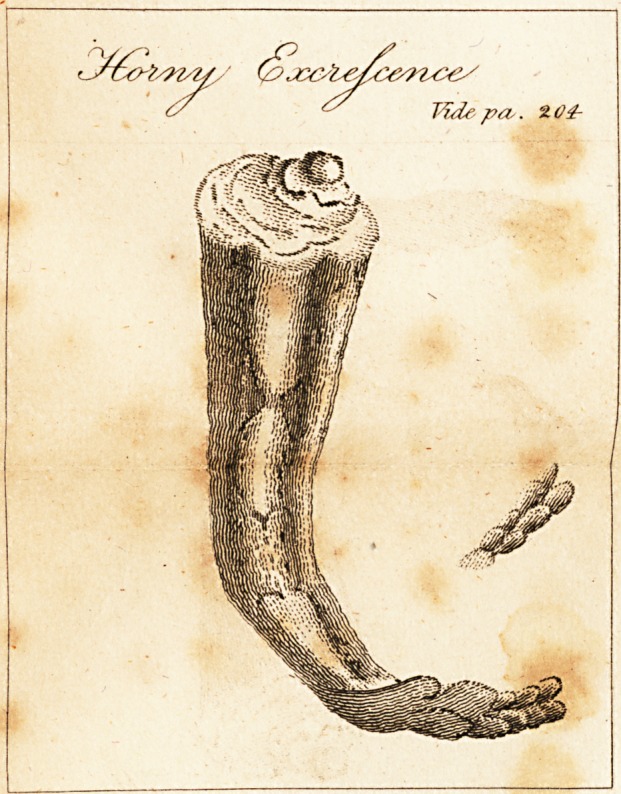# Mr. Edgar's Case of Excrescence on the Scrotum

**Published:** 1804-09-01

**Authors:** Thomas Edgar

**Affiliations:** Fakenham, Norfolk


### *To the Editors of the Medical and Physical Journal.*

GENTLEMEN,

THE following Case having lately occurred to me, I doubt not but it will be acceptable to the Naturalist, and the Profession in general; as such, I send it (with a drawing) for your approbation, and request its insertion in the Med. Journal.

I am, &c.

THOMAS EDGAR.

*Fakenham, Norfolk, June* 14, 1804.

---

MATHEW NOBES, of Stibbard, in this neighbourhood, a chimney-sweeper, two years and a half since, observed an excrescence about the size of, and in appearance like, a wart upon the inferior part of his scrotum; it was, to the best of his recollection, unattended with any humour or inflammation, nor was it preceded by any injury to that part. It continued increasing in size and hardness until it became a perfect horn, as represented in the Drawing. *(See plate.)* This elongation of the epidermis is composed of lamellæ disposed in a longitudinal direction.

From its inconvenient position, and the pain it gave him when in exercise, more especially so in climbing up a chimney, (for in either case he turned the point of the horn upwards) he applied to me for its removal. It was taken off on the 11th of May last; a tolerable bleeding followed, (which was stopped by compressure) the edges of the wound were retained in contact by three ligatures, and the wound was soon cicatrized. The testicle and its vaginal coat were unaffected.

The circumference of its base is two inches and a half; at its apex, three-quarters of an inch; and three inches and and a half in length. He twice cut off a piece the size of that which is shewn in the Drawing, as detached from the horn.

Doctor Monro, during his Lectures, exhibits a straight horn, about four inches in length, taken from the forehead of a woman.

## Figures and Tables

**Figure f1:**